# Periodontitis Is Associated with Endothelial Dysfunction in a General Population: A Cross-Sectional Study

**DOI:** 10.1371/journal.pone.0084603

**Published:** 2013-12-26

**Authors:** Birte Holtfreter, Klaus Empen, Sven Gläser, Roberto Lorbeer, Henry Völzke, Ralf Ewert, Thomas Kocher, Marcus Dörr

**Affiliations:** 1 Unit of Periodontology, Department of Restorative Dentistry, Periodontology, and Endodontology, University Medicine, University of Greifswald, Greifswald, Germany; 2 Department of Internal Medicine, University Medicine, University of Greifswald, Greifswald, Germany; 3 Institute for Community Medicine, SHIP/Clinical-Epidemiological Research, University Medicine, University of Greifswald, Greifswald, Germany; 4 German Centre for Cardiovascular Research (DZHK), partner site Greifswald, Greifswald, Germany; The Ohio State Unversity, United States of America

## Abstract

A large body of evidence underlines an association between periodontal disease and cardiovascular disease. In contrast, data on its relation with endothelial dysfunction as a marker of early subclinical atherosclerosis is inconclusive and limited to patient-cohort studies. We therefore investigated the association between periodontal disease and flow-mediated dilation of the brachial artery (FMD) as a measure of endothelial dysfunction in a general population, and also addressed a possible mediation via inflammation. The study population comprised 1,234 subjects (50.5% men) aged 25–85 years from the 5-year follow-up of the Study of Health in Pomerania, a population-based cohort study. Clinical attachment loss (CAL) and pocket probing depth (PPD) as measures of periodontal disease were assessed half-mouth at four sites per tooth. Subjects were classified according to the periodontitis case definition proposed by Tonetti and Claffey (2005). Measurements of FMD and nitroglycerin-mediated dilation (NMD) were performed using standardized ultrasound techniques. High-sensitive C-reactive protein, fibrinogen and leukocyte count were measured. Fully adjusted multivariate linear regression analyses revealed significant associations of the percentage of sites with PPD ≥6 mm with FMD (p_trend_=0.048), with subjects within the highest category having a 0.74% higher FMD compared to subjects within the lowest category (p<0.05). Consistently, FMD values increased significantly across categories of the percentage of sites with CAL ≥6 mm (p_trend_=0.01) and the periodontitis case definition (p_trend_=0.006). Restrictions to subjects without antihypertensive or statin medication or current non-smokers confirmed previous results. Systemic inflammation did not seem to mediate the relation. Both PPD and CAL were not consistently associated with NMD. In contrast to previous studies, high levels of periodontal disease were significantly associated with high FMD values. This association was not mediated via systemic inflammation. This study revives the discussion on whether and how periodontitis contributes to endothelial dysfunction.

## Introduction

The association between periodontal disease and cardiovascular disease (CVD) is supported by a large body of evidence [1,2,3,4]. Although observational studies suggest that such an association is independent of known confounders, a causative relationship could not be substantiated so far [2]. A potential limitation of previous clinical studies suggesting such a relationship may be confounding by common risk factors of both periodontal disease and cardiovascular disease such as smoking, socioeconomic status, and other cardiovascular risk factors [4].

Periodontitis is a chronic infectious disease involving gingival tissues, the periodontal ligament and the alveolar bone. It is accompanied by increased low grade inflammation and transient bacteremia [5,6,7]. Low grade inflammation, reflected as increased levels of C-reactive protein (CRP) and other inflammatory markers [8,9], is considered as a biologically plausible link between periodontitis and cardiovascular diseases [2]. Particularly inflammation plays an important role in the initiation and progression of atherosclerosis [10,11] and may contribute to the development of coronary heart disease [8,12,13].

The development of atherosclerotic diseases in subjects with cardiovascular risk factors [14] is preceded by endothelial dysfunction. It occurs very early in the pathogenesis of atherosclerosis, before appearance of atheromatous plaques [15] and can be measured as impaired flow-mediated dilation (FMD) [16]. Various studies have shown that low FMD values predict future cardiovascular events independently of cardiovascular risk factors [17,18,19].

Current knowledge on the association between periodontitis and endothelial dysfunction as a marker of subclinical atherosclerosis is mainly based on small comparative and treatment studies. Two studies observed impaired endothelial function in patients with periodontitis compared with controls [20,21,22]. Though the majority of studies reported beneficial effects of periodontal treatments on endothelial function as measured by FMD [21,23,24], evidence proving the cardiovascular benefit of preventive periodontal care or therapeutic interventions is insufficient [4]. 

Summarizing, evidence for an association between periodontitis and endothelial dysfunction is limited and large-scaled observational studies are still missing. Also, potential confounders and particularly the potential role of inflammation as a mediator of this association have not been addressed adequately so far. Therefore, we investigated the association of periodontal disease with endothelial dysfunction in the population-based Study of Health in Pomerania (SHIP) applying different periodontal disease definitions and one periodontal case definition [24]. As a secondary analysis, we evaluated a possible mediation via inflammatory markers. 

## Materials and Methods

### Study of Health in Pomerania

The Study of Health in Pomerania (SHIP) is a longitudinal population-based study in West Pomerania, a northeast area in Germany, with baseline examinations (SHIP-0) conducted in 1997-2001 [25,26]. A two-stage cluster sampling adopted from the World Health Organization Monitoring of Trends and Determinants in Cardiovascular Disease (MONICA) Project [27] yielded twelve 12-year age strata (20-79 years) for both genders, each including 292 subjects. Only Caucasian subjects with German citizenship and main residency in this area were randomly sampled. The net sample included 6267 eligible subjects of whom 4308 subjects participated (response 68.8%). The first follow-up examination (SHIP-1) was conducted about five years after baseline and comprised 3300 subjects. 

### Ethics Statement

The study protocol was approved on the 12/12/2001 by the local Ethics committee of the University of Greifswald (Registration number: III UV 73/01) and all participants gave informed written consent. 

### Covariates

SHIP-1 examinations comprised a computer-aided personal interview on socio-demographic characteristics, health-related behavior, and self-reported medical history. We considered age and gender. School education was defined as <10, 10, or >10 years. Smoking status was assessed using the following questions [28]: “Have you ever smoked cigarettes? If “yes”, “Are you currently smoking cigarettes?”. Based on these questions, smoking status was classified as never, former, or current smoker [29]. Current use of medication was recorded by a computer-aided method using the anatomic, therapeutic, and chemical (ATC) code. The following drugs were considered as antihypertensive medications: antihypertensives (ATC C02), peripheral vasodilators (ATC C04), beta-blockers (ATC C07), calcium antagonists (ATC C08), and medications affecting the renin-angiotensin-system (ATC C09). Statins medication was recorded (ATC C10). Diabetes was defined via self-reported physician’s diagnosis and anti-diabetic medication (ATC A10).

Waist circumference was measured in accordance with the WHO standards and equals the distance (in centimeters) between the narrowest place between the last rib and the highest part of the abdomen. After a five-minute rest period, systolic and diastolic blood pressure (BP) was measured three times in the right arm of seated subjects using a digital blood pressure monitor (HEM-705CP, Omron Corporation, Tokyo, Japan). Each period was followed by a three-minute rest period. The second and third measurements were averaged giving the mean diastolic and systolic BP. Self-reported anti-hypertensive medication within the last twelve months was assessed in the interview. Hypertension was defined if at least one of the following conditions was fulfilled: systolic BP ≥140 mmHg, diastolic BP ≥90 mmHg, or hypertensive medication.

Laboratory measures were determined from non-fasting blood samples drawn from the cubital vein in the supine position. Low-density (LDL-C) and high-density lipoprotein cholesterol (HDL-C) levels were determined enzymatically using a biochemistry autoanalyzer (EPOS Analyzer 5060, Eppendorf, Hamburg, Germany). High sensitive (hs)-CRP was measured in serum by particle-enhanced immunonephelometry (hs-CRP kit, Dade Behring Inc., Eschborn, Germany) with a test sensitivity of 0.2 mg/L. Leukocyte counts were measured using the impedance measurement method (CoultersMaxMt, Coulter Electronics, Miami, FL, USA). Plasma fibrinogen concentrations were assayed according to Clauss using an Electra 1600 analyzer (Instrumentation Laboratory, Barcelona, Spain). 

### Periodontal variables

Periodontal examinations were done according to the half-mouth method, alternating on the left or right side, excluding third molars. A periodontal probe was used (PCP-2, Hu-Friedy, Chicago, IL, USA). Measurements were assessed at four sites (mesiobuccal, midbuccal, distobuccal, and midlingual/midpalatinal). Pocket probing depth (PPD) equals the distance between pocket base and gingival margin. Clinical attachment loss (CAL) equals the distance between the cemento-enamel junction (CEJ) and the pocket base. Where the determination of the CEJ was indistinct (e.g. wedge-shaped defects, fillings, or crown margins), CAL was not recorded. Measurements were mathematically rounded to the whole millimeter. In edentulous subjects no periodontal measurements were recorded.

Various periodontal disease definitions and one periodontitis case definition [24] were evaluated. First, we chose two periodontal disease definitions, which showed the highest gender-specific correlation to common carotid artery intima-media thickness in a previous publication [30]. These comprehend the percentage of sites with CAL ≥6 mm and the percentage of sites with PPD ≥6 mm. Second, subjects were classified according to periodontitis case definition proposed at the 5th European Workshop in Periodontology (referred to as EWP periodontitis case definition) [24], which involves two categories of periodontitis. A “sensitive” (moderate) case was defined by the presence of proximal CAL of ≥3 mm in ≥2 non-adjacent teeth. A “specific” (severe) case was defined by the presence of proximal CAL of ≥5 mm in ≥30% of teeth. Third, mean CAL and mean PPD values were calculated (see Supplement).

Dental examinations were conducted by calibrated and licensed dentists [31]. Half-yearly calibration exercises were performed on subjects not associated with the study. For periodontal assessments intra-class correlations ranged between 0.82 and 0.91 per examiner. Inter-class correlation was 0.84 relative to CAL.

### Endothelial function

FMD of the brachial artery was assessed according to the guidelines of the International Brachial Artery Reactivity Task Force [32], applying the forearm inflation method as described previously [33,34]. This method has also been applied in other large-scaled epidemiological studies including, for example, the Framingham Heart Study [35,36], the Hoorn Study [37] as well as in a large randomized clinical trial evaluating periodontal treatment effects on endothelial function [38]. In contrast to upper arm inflation, which is known to induce greater vasodilation, lower arm inflation has the advantage of precluding the potential contribution of ischemia of the brachial artery itself and being technically less challenging [32]. 

In brief, FMD of the brachial artery was assessed by measuring the increase of the brachial artery diameter during reactive hyperemia after transient forearm ischemia. The brachial artery was visualized using a 10 MHz linear array transducer (Cypress, Siemens, Erlangen, Germany). Ultrasonography was performed in a dark and quiet room. The participants lay quietly for 10 minutes before measurements. A blood pressure cuff was placed around the right forearm 5 cm distally from the right antecubital crease. B-mode longitudinal images of the brachial artery were obtained at the level of the antecubital fossa. After marking the optimal position of the transducer, baseline images of the brachial artery were digitally stored. Arterial flow to the forearm was interrupted by insufflation of the forearm cuff for 5 minutes by 200 mm Hg or 50 mm Hg above systolic blood pressure, whichever was highest. Exactly one minute after cuff deflation, B-mode longitudinal images of the brachial artery were obtained for FMD measurements. After FMD measurements, volunteers laid quietly for 10 minutes to allow the diameters of the brachial arteries to return to baseline levels. Thereafter, nitrate-mediated dilation (NMD) was measured 3 min after sublingual administration of nitroglycerin (400 µg) in 1176 subjects (503 women).

All measurements of brachial artery diameters were performed offline by sonographers and were subjected to strict quality management. Detailed information is given elsewhere [33]. End-diastolic vessel diameters were measured from the anterior to the posterior M-line (i.e., the interface between the media and adventitia) of the vessel wall. Diameters were calculated from the average of three measurements of four consecutive cardiac cycles. Absolute FMD and NMD were calculated by subtracting baseline vessel diameter from post-ischemia and post-nitroglycerin vessel diameters, respectively. Relative changes were expressed as the percentage of absolute FMD and NMD to baseline diameters. 

### Study sample

Between March 2003 and October 2006 1787 subjects (54%) of the SHIP-1 population volunteered for measuring FMD of the brachial artery. Exclusion criteria comprised equipment malfunction (n=36), any medical contraindication (n=4), and hypotension with systolic blood pressure below 100 mmHg (n=15). For 214 FMD examinations reading was not feasible due to poor image quality, resulting in 1518 subjects with readable FMD examinations. Furthermore, 85 subjects with self-reported liver or kidney disease and 140 subjects without PPD data were excluded. Further, we excluded 55 subjects with missing data for inflammatory variables (hs-CRP (n=45), leukocyte count (n=8), and fibrinogen (n=2)), missing confounder data (n=1), or hs-CRP >30 mg/l (n=3). For analyses based on PPD data only, 1234 subjects (50.5% men) were included. Of these, 59 subjects had no CAL data mainly due to crowns, leaving 1175 subjects for analyses of CAL. As the EWP periodontitis case definition criteria necessitates the presence of CAL measurements from at least two teeth [24], subjects with less than two measurements of CAL were excluded, leaving 1136 subjects for analysis.

### Statistical Analyses

Descriptive statistics comprised evaluation of medians with 25% and 75% quantiles for continuous data and assessment of percentage distributions for categorical data. Differences in continuous and categorical data were determined using χ^2^ tests and Mann-Whitney-U-tests, respectively. 

Multivariable linear regression models were run to assess the associations between periodontal disease, hs-CRP, and FMD/NMD. Because the percentages of sites with CAL/PPD ≥6 mm were highly skewed and zero-inflated, both variables were categorized as follows: 0, 0 and ≤median of non-zero values and median of non-zero values. Tertile distributions were determined for mean CAL/PPD. For sensitivity analyses, first- and second-order fractional polynomials [39] were used to compare linear and non-linear forms for exposure variables. To check appropriateness of model assumptions, distributions of residuals were graphically assessed and found to be unsuspicious. All models were adjusted for time between core and FMD examination, age (10-year-categories), gender, education, smoking status, waist circumference, diabetes, HDL-C, LDL-C, and presence of hypertension. Adjusted means with their 95% confidence intervals (CI) were calculated. To assess the role of inflammation as a putative mediator of the association between periodontitis and endothelial dysfunction, hs-CRP was included in full models and relative changes in estimate for periodontal disease measures were determined (Model 4).

For sensitivity analyses, we restricted analyses (i) to subjects without antihypertensive or statin medication to eliminate the possibility that antihypertensive medication might have affected the association of periodontal diseases or hs-CRP with FMD/NMD and (ii) to current non-smokers, because previous smoking may have severely affected FMD measurements. 

To check for possible selection bias introduced by exclusion of subjects (i.e., non-participants of FMD examination, defined as subjects with data on mean PPD and covariates as listed in Table 1, but without FMD data (n=1225)), we applied inverse probability weighting according to major variables of difference (i.e. age, gender, school education, diabetes, hs-CRP, antihypertensive medication, leukocyte counts, diastolic blood pressure, number of teeth, mean PPD) between the study population and non-participants in further sensitivity analyses [40]. 

**Table 1:** Characteristics of the study population and potentially eligible non-participants of the FMD examination.

	All participants (N=1234)	Non-participants (N=1225)	P *
Age, years			
25-34	152 (12.3%)	184 (15.0%)	
35-44	293 (23.7%)	250 (20.4%)	
45-54	309 (25.0%)	243 (19.8%)	
55-64	283 (22.9%)	258 (21.1%)	
65-74	163 (13.2%)	201 (16.4%)	
75-88	34 (2.8%)	89 (7.3%)	<0.001
Male gender	623 (50.5%)	552 (45.1%)	0.007
*Socio-economic and behavioral variables*			
School education			
<10 years	312 (25.3%)	399 (32.6%)	
10 years	665 (53.9%)	604 (49.3%)	
>10 years	257 (20.8%)	222 (18.1%)	<0.001
Smoking status			
Never smoker	533 (43.2%)	521 (42.5%)	
Former smoker	388 (31.4%)	355 (29.0%)	
Current smoker	313 (25.4%)	349 (28.5%)	0.17
Diabetes #	82 (6.7%)	117 (9.6%)	0.008
Waist circumference, cm	92.0 (82.6; 101.5)	91.1 (81.0; 101.1)	0.15
Body Mass Index, kg/m^2^	27.3 (24.6; 30.6)	27.1 (24.1; 30.8)	0.38
*Laboratory variables*			
LDL-C, mmol/L **	3.5 (2.8; 4.2)	3.4 (2.8; 4.2)	0.54
HDL-C, mmol/L **	1.1 (0.9; 1.4)	1.2 (0.9; 1.5)	0.26
High sensitive CRP, mg/l	1.2 (0.6; 2.7)	1.4 (0.7; 3.0)	0.002
Leukocytes, Gpt/L	6.5 (5.4; 7.6)	6.7 (5.6; 7.9)	<0.001
Fibrinogen (Clauss), g/l	3.0 (2.6; 3.6)	3.1 (2.6; 3.6)	0.12
Systolic blood pressure, mmHg	129 (118; 141)	130 (117; 143)	0.39
Diastolic blood pressure, mmHg	82 (76; 89)	81 (74; 88)	0.005
Hypertension †	571 (46.3%)	589 (48.1%)	0.37
*Medications – Use of…*			
Antihypertensive medication	366 (30.0%)	445 (36.3%)	<0.001
Statins	112 (9.1%)	129 (10.5%)	0.23
*Variables assessing endothelial dysfunction*			
Time between FMD and core examination, months	0.5 (0; 2.3)	-	-
Baseline diameter A. brachialis, mm	3.86 (3.36; 4.41)	-	
Post-occlusion diameter A. brachialis, mm	4.06 (3.53; 4.60)	-	
FMD, %	4.65 (2.37; 7.21)	-	
NMD, % ‡	13.58 (9.35; 18.87)	-	
*Dental variables*			
Percentage of sites with PPD ≥6 mm, %	0 (0; 1.8)	0 (0; 1.9)	0.20
Percentage of sites with CAL ≥6 mm, % §	0 (0; 6.3)	0 (0; 11.4)	<0.001
Mean PPD, mm	2.09 (1.83; 2.57)	2.19 (1.87; 2.75)	0.005
Mean CAL, mm §	1.88 (0.83; 3.10)	2.19 (1.02; 3.60)	<0.001
EWP periodontitis case definition ‖			
No periodontitis	430 (37.8%)	363 (32.5%)	
Sensitive definition (moderate periodontitis)	435 (38.3%)	408 (36.6%)	
Specific definition (severe periodontitis)	271 (23.9%)	345 (30.9%)	<0.001
Number of teeth in dentates	23 (18; 26)	22 (15; 26)	<0.001

Data are presented as number (percentage) or median (25%; 75% quantiles). LDL-C, Low-density lipoprotein cholesterol; HDL-C, High-density lipoprotein cholesterol; CRP, C-reactive protein; FMD, flow-mediated dilation; NMD, nitrate-mediated dilation; PPD, pocket probing depth; CAL, clinical attachment loss. * χ^2^ test (categorical data) or Mann-Whitney-U-test (continuous data); ** non-fasting blood samples; # self-reported physician’s diagnosis or medication (ATC code A10); † systolic blood pressure ≥140 mmHg or diastolic blood pressure ≥90 mmHg or self-reported use of antihypertensive medication within last 12 months; ‡ N=951; § N=1175; ‖ N=1136.

A p<0.05 was considered statistically significant. All statistical analyses were performed with STATA/SE 12.1 [41].

## Results

### Study population

Compared to non-participants, study participants had lower hs-CRP and leukocyte counts (Table 1). They were younger, were more often males, were more commonly highly educated and had less often diabetes mellitus. They had lower diastolic blood pressures and used less often antihypertensive medication relative to non-participants. There were no differences in smoking status, waist circumference, LDL-C, HDL-C, fibrinogen, and systolic blood pressure. For participating subjects, median time between core and FMD examination was 0.5 months.

### Association between PPD measures and FMD

After adjustment for age, gender, and time between core and FMD examination (Table 2, Model 1), the percentage of sites with PPD ≥6 mm was associated with FMD with borderline significance (p_trend_=0.09). In the fully adjusted model (Model 3) FMD increased significantly across exposure groups (p_trend_=0.048) with subjects within the third category having a 0.74% higher FMD value compared to the reference category (p<0.05). Inclusion of hs-CRP into the full model changed coefficients for the percentage of sites with PPD ≥6 mm by less than 1% (Model 4).

**Table 2:** Association between percentage of sites with pocket probing depth ≥6 mm (exposure) and FMD (dependent variable) in all subjects, in subjects without antihypertensive or statin medication, or in current non-smokers.

	Percentage of sites with pocket probing depth ≥6 mm			
	0% (ref.)	1.7-5.8%	6.2-66.7%	P_trend_
*All subjects (N=1234)*				
Model 1	5.08 (4.84; 5.31)	5.13 (4.64; 5.61)	5.69 (5.07; 6.30)	0.09
Model 2	5.06 (4.82; 5.30)	5.10 (4.62; 5.58)	5.80 (5.18; 6.42) *	0.046
Model 3	5.06 (4.82; 5.30)	5.09 (4.61; 5.57)	5.80 (5.18; 6.42) *	0.048
Model 4	5.07 (4.83; 5.31)	5.05 (4.56; 5.53)	5.81 (5.19; 6.43) *	0.06
*Subjects without antihypertensive or statin medication (N=832)*				
Model 1	5.47 (5.18; 5.76)	5.58 (4.97; 6.20)	6.27 (5.35; 7.19)	0.12
Model 2	5.46 (5.16; 5.75)	5.56 (4.94; 6.17)	6.42 (5.49; 7.35)	0.07
Model 3	5.46 (5.16; 5.75)	5.55 (4.94; 6.17)	6.42 (5.50; 7.35)	0.07
Model 4	5.47 (5.17; 5.76)	5.48 (4.85; 6.11)	6.45 (5.53; 7.38) *	0.08
*Current non-smokers (N=921)*				
Model 1	5.08 (4.81; 5.35)	5.08 (4.55; 5.62)	5.95 (5.18; 6.72) *	0.07
Model 2	5.08 (4.81; 5.35)	5.09 (4.55; 5.63)	5.92 (5.15; 6.70) *	0.08
Model 3	5.09 (4.81; 5.36)	5.06 (4.53; 5.60)	5.95 (5.18; 6.72) *	0.08
Model 4	5.09 (4.83; 5.36)	5.00 (4.46; 5.54)	5.96 (5.19; 6.74) *	0.09

Adjusted means for FMD with 95% CIs are given. P_trend_: p for linear trend; FMD, flow-mediated dilation. Model 1: adjusted for time between core and FMD examination, age (10-year-categories), and sex; Model 2: Model 1 plus school education (three categories) and smoking status (three categories); Model 3 – fully adjusted model: Model 2 plus diabetes, waist circumference, High-density lipoprotein cholesterol, Low-density lipoprotein cholesterol, and hypertension. Model 4: fully adjusted model 3 plus hs-CRP. * p<0.05 versus reference category (ref.). Numbers within categories were 910, 170, and 154 for all subjects, 624, 123, and 85 for subjects without antihypertensive or statin medication, and 689, 132, and 100 for current non-smokers.

Several sensitivity analyses were performed (Table 2). First, restricting subjects to those without antihypertensive or statin medication, the association was non-significant (p_trend_=0.07), but consistent with previous results regarding direction of the association. Second, restricting analyses to current non-smokers revealed that subjects within the third category had a 0.86% higher FMD value compared to the reference category (p<0.05). Results were also consistent regarding the direction of the association if we i) included only subjects with hs-CRP ≤10 mg/l (p_trend_=0.03), ii) restricted subjects to those having at least four teeth, thereby increasing robustness of periodontal estimates (p_trend_=0.07), and iii) exchanged the exposure by mean PPD (p_trend_=0.08, Table S1). 

### Association between CAL measures and FMD

After adjustment for age, gender, and time between core and FMD examination (Table 3, Model 1), the percentage of sites with CAL ≥6 mm was linearly associated with FMD (p_trend_=0.02). After full adjustment (Model 3) FMD increased across increasing exposure categories (p_trend_=0.01) with subjects within the third category having a 0.7% higher FMD value compared to the reference category (p<0.05). Inclusion of hs-CRP into the full model changed coefficients for the percentage of sites with CAL ≥6 mm by less than 1% (Model 4).

**Table 3:** Association between percentage of sites with clinical attachment loss ≥6 mm (exposure) and FMD (dependent variable) in all subjects, in subjects without antihypertensive or statin medication, or in current non-smokers.

	Percentage of sites with clinical attachment loss ≥6 mm			
	0% (ref.)	1.7-12.5%	13.0-100%	P_trend_
*All subjects (N=1175)*				
Model 1	5.07 (4.81; 5.34)	5.36 (4.83; 5.90)	5.70 (5.24; 6.16) *	0.02
Model 2	5.05 (4.78; 5.32)	5.39 (4.85; 5.93)	5.75 (5.29; 6.20) *	0.01
Model 3	5.05 (4.78; 5.32)	5.39 (4.85; 5.93)	5.75 (5.29; 6.21) *	0.01
Model 4	5.06 (4.79; 5.33)	5.38 (4.83; 5.92)	5.72 (5.27; 6.18) *	0.02
*Subjects without antihypertensive or statin medication (N=806)*				
Model 1	5.50 (5.18; 5.82)	5.71 (5.03; 6.40)	6.24 (5.55; 6.93)	0.08
Model 2	5.47 (5.14; 5.79)	5.75 (5.06; 6.44)	6.34 (5.66; 7.02) *	0.04
Model 3	5.47 (5.14; 5.79)	5.77 (5.06; 6.47)	6.32 (5.64; 7.00) *	0.04
Model 4	5.48 (5.16; 5.80)	5.73 (5.02; 6.44)	6.31 (5.64; 6.99) *	0.053
*Current non-smokers (N=868)*				
Model 1	5.05 (4.76; 5.35)	5.45 (4.82; 6.08)	5.83 (5.30; 6.36) *	0.01
Model 2	5.07 (4.77; 5.38)	5.42 (4.79; 6.05)	5.79 (5.26; 6.32) *	0.03
Model 3	5.07 (4.77; 5.38)	5.42 (4.79; 6.05)	5.79 (5.26; 6.32) *	0.03
Model 4	5.08 (4.77; 5.38)	5.42 (4.79; 6.05)	5.78 (5.26; 6.31) *	0.03

Adjusted means for FMD with 95% CIs are given. P_trend_: p for linear trend; FMD, flow-mediated dilation. Model 1: adjusted for time between core and FMD examination, age (10-year-categories), and sex; Model 2: Model 1 plus school education (three categories) and smoking status (three categories); Model 3 – fully adjusted model: Model 2 plus diabetes, waist circumference, High-density lipoprotein cholesterol, Low-density lipoprotein cholesterol, and hypertension. Model 4: fully adjusted model 3 plus hs-CRP. * p<0.05 versus reference category (ref.). Numbers within categories were 737, 223, and 215 for all subjects, 554, 142, and 110 for subjects without antihypertensive or statin medication, and 545, 163, and 160 for current non-smokers.

In subjects without antihypertensive or statin medication (Table 3), FMD increased significantly across exposure categories (p_trend_=0.04) and was highest in the most severely diseased. Second, restricting analyses to current non-smokers revealed that subjects within the third category had a 0.72% higher FMD value compared to the reference category (p<0.05). Results were also consistent if we i) included only subjects with hs-CRP ≤10 mg/l (p_trend_=0.01), ii) restricted subjects to those having at least four teeth (p_trend_=0.03), and iii) exchanged the exposure by mean CAL (p_trend_=0.02, Table S2). 

### Association between the EWP periodontitis case definition and FMD

In fully adjusted models (Table 4, Model 3) FMD was significantly higher in sensitive /moderate (5.38%) and specific/severe cases (5.71%) compared to subjects with no periodontitis (4.81%, p_trend_=0.006). Inclusion of hs-CRP into full models did not change the coefficients relevantly. Significant associations were confirmed if subjects were restricted to i) those without antihypertensive or statin medication (p_trend_=0.01) or ii) current non-smokers (p_trend_=0.02). 

**Table 4:** Association between the EWP periodontitis case definition (exposure) and FMD (dependent variable) in all subjects, in subjects without antihypertensive or statin medication, or in current non-smokers.

	EWP periodontitis case definition			
	No periodontitis (ref.)	Sensitive case	Specific case	P_trend_
*All subjects (N=1136)*				
Model 1	4.85 (4.49; 5.21)	5.38 (5.01; 5.76)	5.64 (5.16; 6.11) *	0.01
Model 2	4.82 (4.46; 5.18)	5.38 (5.00; 5.75) *	5.70 (5.22; 6.18) **	0.007
Model 3	4.81 (4.45; 5.17)	5.38 (5.00; 5.75) *	5.71 (5.23; 6.18) **	0.006
Model 4	4.83 (4.47; 5.19)	5.38 (5.01; 5.75) *	5.67 (5.19; 6.15) *	0.009
*Subjects without antihypertensive or statin medication (N=782)*				
Model 1	5.22 (4.81; 5.63)	5.83 (5.35; 6.30)	6.18 (5.48; 6.87) *	0.02
Model 2	5.19 (4.77; 5.60)	5.82 (5.35; 6.29)	6.27 (5.57; 6.97) *	0.01
Model 3	5.18 (4.77; 5.60)	5.83 (5.35; 6.30)	6.27 (5.56; 6.97) *	0.01
Model 4	5.21 (4.79; 5.62)	5.82 (5.36; 6.28)	6.22 (5.51; 6.93) *	0.02
*Current non-smokers (N=834)*				
Model 1	4.91 (4.48; 5.33)	5.26 (4.85; 5.67)	5.92 (5.35; 6.49) **	0.01
Model 2	4.93 (4.49; 5.36)	5.27 (4.86; 5.68)	5.88 (5.31; 6.44) *	0.02
Model 3	4.92 (4.49; 5.36)	5.27 (4.85; 5.68)	5.88 (5.32; 6.45) *	0.02
Model 4	4.94 (4.50; 5.37)	5.26 (4.86; 5.67)	5.86 (5.29; 6.43) *	0.03

Adjusted means for FMD with 95% CIs are given. P_trend_: p for linear trend; FMD, flow-mediated dilation. Model 1: adjusted for time between core and FMD examination, age (10-year-categories), and sex; Model 2: Model 1 plus school education (three categories) and smoking status (three categories); Model 3 – fully adjusted model: Model 2 plus diabetes, waist circumference, High-density lipoprotein cholesterol, Low-density lipoprotein cholesterol, and hypertension. Model 4: fully adjusted model 3 plus hs-CRP. * p<0.05, ** p<0.01 versus reference category (ref.). Numbers within categories were 430, 435, and 271 for all subjects, 342, 290, and 150 for subjects without antihypertensive or statin medication, and 311, 329, and 194 for current non-smokers.

### Association between hs-CRP and FMD

To further address the role of systemic inflammation as a potential mediator of the link between periodontitis and endothelial dysfunction, we also evaluated the association between hs-CRP and FMD. In the fully adjusted model (Table 4, Model 3), hs-CRP was non-significantly related to FMD (adjusted means: 5.04, 4.93, and 5.50%; p_trend_=0.09). In sensitivity analyses results were consistent if we excluded either subjects with antihypertensive or statin medication (p_trend_=0.32, Table 5), current smokers (p_trend_=0.04, Table 5), or subjects with hs-CRP values >10 mg/l (p_trend_=0.22). Associations were consistently non-significant if fibrinogen or leukocyte count were considered as exposures (data not shown).

**Table 5:** Association between hs-CRP (tertiles, exposure) and FMD (dependent variable) in all subjects, in subjects without antihypertensive or statin medication, or in current non-smokers.

	hs-CRP			
	0.17-0.816 mg/l (ref.)	0.82-2.08 mg/l	2.09-29.1 mg/l	P_trend_
*All subjects (N=1234)*				
Model 1	5.10 (4.74; 5.47)	4.91 (4.57; 5.25)	5.46 (5.10; 5.82)	0.18
Model 2	5.11 (4.74; 5.48)	4.92 (4.58; 5.26)	5.45 (5.09; 5.80)	0.20
Model 3	5.04 (4.67; 5.42)	4.93 (4.59; 5.27)	5.50 (5.14; 5.87)	0.09
*Subjects without antihypertensive or statin medication (N=832)*				
Model 1	5.58 (5.14; 6.03)	5.24 (4.82; 5.65)	5.91 (5.44; 6.38)	0.38
Model 2	5.59 (5.13; 6.04)	5.24 (4.82; 5.65)	5.90 (5.43; 6.37)	0.39
Model 3	5.56 (5.10; 6.03)	5.23 (4.81; 5.65)	5.94 (5.46; 6.42)	0.32
*Current non-smokers (N=921)*				
Model 1	5.10 (4.68; 5.52)	4.87 (4.48; 5.27)	5.54 (5.14; 5.94)	0.14
Model 2	5.12 (4.69; 5.54)	4.87 (4.47; 5.26)	5.53 (5.13; 5.93)	0.17
Model 3	5.00 (4.56; 5.43)	4.87(4.47; 5.26)	5.64 (5.23; 6.06) *	0.04

Adjusted means for FMD with 95% CI are given. P_trend_: p for linear trend; FMD, flow-mediated dilation. Model 1: adjusted for time between core and FMD examination, age (10-year-categories), and sex; Model 2: Model 1 plus school education (three categories) and smoking status (three categories); Model 3 – fully adjusted model: Model 2 plus diabetes, waist circumference, High-density lipoprotein cholesterol, Low-density lipoprotein cholesterol, and hypertension. * p<0.05 versus reference category (ref.). Numbers within categories were 413, 410, and 411 for all subjects, 310, 269, and 253 for subjects without antihypertensive or statin medication, and 304, 302, and 315 for current non-smokers.

All findings did not change substantially after employment of first- and second-degree multivariate fractional polynomial models and inverse probability weighting.

## Discussion

To our knowledge, this is the first population-based study evaluating the association between periodontitis and endothelial dysfunction as measured by FMD. High levels of periodontal disease severity were significantly associated with high FMD levels, but hs-CRP was not associated with FMD levels. These findings are in contrast to the majority of previous investigations, which were mainly based on patient-cohort studies.

### Association between periodontitis and FMD

In contrast to our expectations, higher levels of periodontal disease were associated with higher FMD values. Several sensitivity analyses confirmed these results. Our findings are contradictory to previous observational studies. Three observational studies reported that endothelial function was decreased in patients with periodontitis compared to healthy controls [20,21,22]. In line with this, periodontal treatment was reported to be accompanied by an improvement of endothelial function. Tonetti et al. (2007) conducted a randomized clinical trial comparing intensive periodontal treatment with community-based treatment in 120 patients with severe periodontitis. Periodontal treatment resulted in short-term increase of inflammatory markers and endothelial dysfunction immediately after the treatment. After 6 months, endothelial function improved parallel to oral health as defined by a reduced number of periodontal pockets or a reduced number of sites with plaque or gingival bleeding [38]. Similar long-term results have also been reported by various smaller treatment studies [21,22,42]. Consistently, beneficial effects of periodontal treatments were also observed for other measures of endothelial function [43,44].

The discrepancies between our and other studies are difficult to explain. Possibly, differences might partly be explained by the population-based origin of our study sample. Participants of SHIP were compromised by comorbidities such as diabetes mellitus, cardiovascular disease, and chronic kidney disease. It is well-established that endothelial function is lowered in the presence of cardiovascular risk factors [35,45]. On the other hand SHIP subjects had less periodontal disease than the subjects in the landmark study of Tonetti [38]. In addition, study participants included in the current analysis had even lower CAL and PPD levels (see Table 1) than the overall SHIP sample. In contrast to SHIP, studies reporting an association between periodontitis and endothelial function or between periodontal treatment and improvement in endothelial function were performed predominantly in otherwise healthy patients with severe periodontitis [20,21,22,23,38,42,43,44]. Thus, evidence is suggesting that the association between periodontitis and endothelial function is more likely to be found in subjects with severe stages of the disease, who on the other hand don’t present many cardiovascular risk factors. In line with this hypothesis, another treatment study found that endothelial function was significantly lowered in patients with severe periodontitis, but not in patients with mild periodontitis compared to controls [20]. Another randomized controlled trial in 50 otherwise healthy subjects with moderate-to-severe chronic periodontitis, however, could not confirm an improvement in peripheral vascular endothelial function after periodontal treatment [46]. 

### Mediation via low grade systemic inflammation

Low grade systemic inflammation was expected to be the major component linking periodontitis and endothelial dysfunction. In a large meta-analyses of case-control and longitudinal treatment studies CRP levels were significantly higher in subjects with periodontitis as compared to controls [8]. Other studies found that subjects with severe periodontitis had increased serum levels of CRP and interleukins [6,47], hyperfibrinogenemia, and moderate leukocytosis [48]. Also in SHIP, concordant findings were reported [49]. It is assumed that once released into the circulation, LPS and cytokines induce systemic effects [47], including increased hepatic CRP production [50] as well as an increased production of acute-phase proteins, pro-coagulant mediators, and more pro-inflammatory cytokines [51]. These in turn may further induce the inflammatory processes leading to endothelial atheroma formation [51]. 

To address the potential aspect of mediation of the link between periodontitis and FMD via low grade systemic inflammation and find an explanation for our unexpected results, we assessed the change in coefficients for periodontal disease measures after inclusion of hs-CRP into full models (Table 2, 3, and 4; Model 4) and evaluated the relation between hs-CRP and FMD (Table 5). Since coefficients for periodontal disease measures changed by less than 1% and hs-CRP was not associated with FMD, systemic inflammation did not seem to mediate the link between periodontal diseases and endothelial function. 

Conflicting results regarding the association between inflammatory markers, including CRP, and endothelial function were also demonstrated by others. In the Framingham Offspring Study FMD was not associated with inflammation markers after accounting for traditional coronary risk factors [36]. In contrast, other studies suggested that elevated CRP levels were associated with impaired FMD [52,53]. Concordantly, the reduction of CRP levels was accompanied by an improvement of endothelial function [52]. However, since these patient cohorts were small and highly selective, including patients with coronary vascular disease [52] or coronary heart disease [53], large-scaled cohort studies are needed to clarify any putative causative link.

In contrast to FMD, NMD is a marker of endothelium independent vasodilation. Considering NMD as the dependent variable, no consistent associations were found if different parameterizations were used (see Supporting Information, Tables S3, S4, and S5). As expected, periodontitis did not affect the endothelium independent vasodilation, but might affect endothelial function via the NO system. 

If our observation was true, periodontitis might reflect an unmeasured aspect of a positive general health factor or health behavior or it might be a surrogate marker for an unmeasured aspect, which mimics the positive relation with endothelial function. However this statement is not supported in periodontal literature. In a recent review, D´Aiuto et al. reported a consistent effect of periodontal therapy in improvement of endothelial-dependent function [54].

### Strengths and limitations

The major strength of this study is its population-based approach including a general German adult population sample with a broad age range. Second, to exclude measurement error during assessment of endothelial function induced by previous smoking, current smokers were excluded in sensitivity analyses. Besides, residual confounding by current smoking intensity was reduced as smoking mainly confounds the association between periodontitis [55,56] and endothelial function [57,58]. However, in current non-smokers, associations between periodontal disease measures and endothelial function sustained positive. Third, addressing quality issues of periodontal variables, dental examiners were a priori trained and certified and intra- and inter-observer variabilities indicated moderate to high accuracy of periodontal measurements. In line with the current literature, periodontitis was significantly associated with HbA1c [59,60] or atherosclerosis measures in a dose dependent manner [61] using SHIP data. Fourth, assessment of endothelial function was performed under strict quality management by standardized protocol and certified staff [33] using forearm ischemia-induced FMD measurement according to the guidelines of the International Brachial Artery Reactivity Task Force [32]. Consequently, FMD values measured in our study may not be directly compared to those of most clinical studies evaluating periodontal treatment effects on endothelial function [20,21,42] since those studies used upper arm inflation. However, the advantage of lower over upper arm inflation is that it precludes the potential contribution of ischemia of the brachial artery itself [32]. In previous studies using the SHIP cohort, endothelial dysfunction was linked to different exposures, including lower serum total and free testosterone levels [34], high plasma aldosterone concentrations and the aldosterone-to-renin ratio [62], increased serum haemoglobin A(1c) levels [63], lower serum Insulin-like growth factor-1 levels [64], and impaired renal function [65].

Furthermore, FMD measurements were potentially susceptible to observer bias. Though ultrasound observers were a priori trained and certified, intra- and inter-observer variabilities indicate some limitations with regard to robustness of FMD measurements [33]. However, systematic observer bias was unlikely, because dental and FMD examinations were performed by separated groups of observers, and none of the groups knew the results of the other examination. Sixth, residual confounding remains a considerable concern, although we comprehensively adjusted for potential confounding.

Some other limitations need to be considered when interpreting the findings of the present study. First, because of its cross-sectional design, no causative relationship could be established. Second, selection bias due to non-participation in FMD measurements may have occurred, because younger and healthier subjects volunteered (Table 1). However, findings remained consistent if inverse probability weighting, a method to evaluate possible selection bias introduced by exclusion of subjects [40], was applied. Third, as full-mouth periodontal recordings are time-consuming and cost intensive, large epidemiological studies often must accept partial recording protocols [66]. In SHIP, periodontal measurements were taken according to the half-mouth method at three sites per tooth, which is known to be associated with an underestimation of periodontal disease severity [67]. Consequently, effect estimates might even be biased towards the null [68]. Finally, the median time between core and FMD examination was 0.5 months. We cannot exclude the possibility that between examinations periodontal treatment was performed. However, it is unlikely that this had relevant effects on periodontal status. Further, adjustment for the time between core and FMD examination did not change exposure coefficients considerably (data not shown).

## Conclusions

In conclusion, we revealed a positive association between periodontitis and endothelial function in a population-based study. The biological plausibility of a periodontitis–FMD association appears remote. The analysis of hs-CRP in the relation between periodontitis and FMD did not help to unravel any biological pathway. Despite the fact that there might be an association between periodontitis and FMD, it may be spurious. Our observation stands in contrast to the majority of previous patient-cohort based studies. In addition, given the broad evidence on the link between periodontitis and cardiovascular diseases [1,3], results are difficult to explain. Thus, the association between periodontitis and endothelial function needs further clarification both in thoroughly designed longitudinal and interventional studies.

## Supporting Information

Table S1 **Association between mean pocket probing depth (tertiles, exposure) and FMD (dependent variable) in all subjects, in subjects without antihypertensive or statin medication, or in current non-smokers.**
(DOCX)

Table S2 **Association between mean clinical attachment loss (tertiles, exposure) and FMD (dependent variable) in all subjects, in subjects without antihypertensive or statin medication, or in current non-smokers.**
(DOCX)

Table S3 **Association between mean pocket probing depth (tertiles, exposure) and NMD (dependent variable).**
(DOCX)

Table S4 **Association between mean clinical attachment loss (tertiles, exposure) and NMD (dependent variable).**
(DOCX)

Table S5 **Association between hs-CRP (tertiles, exposure) and NMD (dependent variable).**
(DOCX)
