# Supplementary material for: Periodontitis Is Associated with Endothelial Dysfunction in a General Population: A Cross-Sectional Study
Source: PLoS One. 2013 Dec 26;8(12):e84603. doi: 10.1371/journal.pone.0084603 (PMC3873439; doi:10.1371/journal.pone.0084603)
Supplement: Table S1 — Association between mean pocket probing depth (tertiles, exposure) and FMD (dependent variable) in all subjects, in subjects without antihypertensive or statin medication, or in current non-smokers. (DOCX) [file pone.0084603.s001.docx]

Table S1. Association between mean pocket probing depth (tertiles, exposure) and FMD (dependent variable) in all subjects, in subjects without antihypertensive or statin medication, or in current non-smokers.

|  | Mean pocket probing depth | | |  |
| --- | --- | --- | --- | --- |
|  | 1.21-1.94 mm (ref.) | 1.94-2.45 mm | 2.45-7.63 mm | P_trend_ |
| *All subjects (N=1234)* | | | | |
| Model 1 | 4.90 (4.57; 5.24) | 5.33 (4.95; 5.70) | 5.27 (4.90; 5.63) | 0.16 |
| Model 2 | 4.87 (4.53; 5.22) | 5.30 (4.93; 5.67) | 5.33 (4.96; 5.69) | 0.08 |
| Model 3 | 4.87 (4.53; 5.21) | 5.30 (4.93; 5.68) | 5.33 (4.96; 5.69) | 0.08 |
| Model 4 | 4.89 (4.55; 5.23) | 5.29 (4.93; 5.66) | 5.32 (4.95; 5.68) | 0.10 |
| *Subjects without antihypertensive or statin medication (N=832)* | | | | |
| Model 1 | 5.37 (4.95; 5.78) | 5.71 (5.24; 6.18) | 5.69 (5.20; 6.19) | 0.31 |
| Model 2 | 5.33 (4.92; 5.75) | 5.69 (5.21; 6.16) | 5.77 (5.26; 6.27) | 0.20 |
| Model 3 | 5.34 (4.92; 5.76) | 5.68 (5.21; 6.16) | 5.77 (5.26; 6.27) | 0.20 |
| Model 4 | 5.37 (4.95; 5.79) | 5.66 (5.20; 6.11) | 5.76 (5.25; 6.26) | 0.25 |
| *Current non-smokers (N=921)* | | | | |
| Model 1 | 4.83 (4.44; 5.21) | 5.32 (4.91; 5.72) | 5.43 (5.00; 5.87) * | 0.045 |
| Model 2 | 4.84 (4.45; 5.23) | 5.31 (4.91; 5.71) | 5.42 (4.99; 5.86) | 0.06 |
| Model 3 | 4.82 (4.43; 5.21) | 5.31 (4.91; 5.72) | 5.44 (5.01; 5.88) * | 0.04 |
| Model 4 | 4.84 (4.46; 5.23) | 5.29 (4.89; 5.69) | 5.44 (5.01; 5.88) | 0.049 |

Adjusted means for FMD with 95% CIs are given. P_trend_: p for linear trend; FMD, flow-mediated dilation. Model 1: adjusted for time between core and FMD examination, age (10-year-categories), and sex; Model 2: Model 1 plus school education (three categories) and smoking status (three categories); Model 3 – fully adjusted model: Model 2 plus diabetes, waist circumference, High-density lipoprotein cholesterol, Low-density lipoprotein cholesterol, and hypertension. Model 4: fully adjusted model 3 plus hs-CRP. * p<0.05 versus reference category (ref.)
