# Supplementary material for: Periodontitis Is Associated with Endothelial Dysfunction in a General Population: A Cross-Sectional Study
Source: PLoS One. 2013 Dec 26;8(12):e84603. doi: 10.1371/journal.pone.0084603 (PMC3873439; doi:10.1371/journal.pone.0084603)
Supplement: Table S2 — Association between mean clinical attachment loss (tertiles, exposure) and FMD (dependent variable) in all subjects, in subjects without antihypertensive or statin medication, or in current non-smokers. (DOCX) [file pone.0084603.s002.docx]

Table S2. Association between mean clinical attachment loss (tertiles, exposure) and FMD (dependent variable) in all subjects, in subjects without antihypertensive or statin medication, or in current non-smokers.

|  | Mean clinical attachment loss | | |  |
| --- | --- | --- | --- | --- |
|  | 0-1.14 mm (ref.) | 1.16-2.58 mm | 2.58-11.86 mm | P_trend_ |
| *All subjects (N=1175)* | | | | |
| Model 1 | 4.95 (4.55; 5.35) | 5.23 (4.82; 5.63) | 5.55 (5.17; 5.94) | 0.05 |
| Model 2 | 4.90 (4.50; 5.31) | 5.20 (4.80; 5.61) | 5.62 (5.23; 6.01) * | 0.02 |
| Model 3 | 4.91 (4.51; 5.32) | 5.19 (4.78; 5.60) | 5.63 (5.24; 6.02) * | 0.02 |
| Model 4 | 4.93 (4.53; 5.34) | 5.21 (4.80; 5.61) | 5.59 (5.20; 5.98) * | 0.04 |
| *Subjects without antihypertensive or statin medication (N=806)* | | | | |
| Model 1 | 5.42 (4.98; 5.87) | 5.70 (5.18; 6.22) | 5.89 (5.34; 6.44) | 0.24 |
| Model 2 | 5.37 (4.92; 5.82) | 5.68 (5.16; 6.20) | 6.00 (5.44; 6.55) | 0.13 |
| Model 3 | 5.38 (4.92; 5.83) | 5.67 (5.15; 6.20) | 5.99 (5.43; 6.55) | 0.13 |
| Model 4 | 5.40 (4.94; 5.85) | 5.70 (5.18; 6.22) | 5.93 (5.37; 6.49) | 0.19 |
| *Current non-smokers (N=868)* | | | | |
| Model 1 | 4.76 (4.29; 5.22) | 5.31 (4.87; 5.75) | 5.74 (5.29; 6.20) ** | 0.006 |
| Model 2 | 4.78 (4.31; 5.25) | 5.32 (4.88; 5.76) | 5.71 (5.26; 6.16) * | 0.01 |
| Model 3 | 4.79 (4.32; 5.27) | 5.29 (4.85; 5.74) | 5.72 (5.27; 6.18) * | 0.01 |
| Model 4 | 4.80 (4.33; 5.27) | 5.31 (4.87; 5.75) | 5.70 (5.25; 6.16) * | 0.01 |

Adjusted means for FMD with 95% CIs are given. P_trend_: p for linear trend; FMD, flow-mediated dilation. Model 1: adjusted for time between core and FMD examination, age (10-year-categories), and sex; Model 2: Model 1 plus school education (three categories) and smoking status (three categories); Model 3 – fully adjusted model: Model 2 plus diabetes, waist circumference, High-density lipoprotein cholesterol, Low-density lipoprotein cholesterol, and hypertension. Model 4: fully adjusted model 3 plus hs-CRP. * p<0.05, ** p<0.01 versus reference category (ref.)
