# Supplementary material for: Periodontitis Is Associated with Endothelial Dysfunction in a General Population: A Cross-Sectional Study
Source: PLoS One. 2013 Dec 26;8(12):e84603. doi: 10.1371/journal.pone.0084603 (PMC3873439; doi:10.1371/journal.pone.0084603)
Supplement: Table S3 — Association between mean pocket probing depth (tertiles, exposure) and NMD (dependent variable). (DOCX) [file pone.0084603.s003.docx]

Table S3. Association between mean pocket probing depth (tertiles, exposure) and NMD (dependent variable).

|  | Mean pocket probing depth | | |  |
| --- | --- | --- | --- | --- |
|  | 1.21-1.94 mm (ref.) | 1.94-2.45 mm | 2.45-7.63 mm | P_trend_ |
| *All subjects (N=951)* | | | | |
| Model 1 | 14.27 (13.5515.00) | 14.76 (14.0915.43) | 14.42 (13.7215.13) | 0.77 |
| Model 2 | 14.15 (13.4414.86) | 14.76 (14.0915.42) | 14.56 (13.8515.27) | 0.44 |
| Model 3 | 14.16 (13.4514.87) | 14.71 (14.0515.36) | 14.60 (13.9015.31) | 0.40 |
| *Subjects without antihypertensive medication (N=597)* | | | | |
| Model 1 | 15.32 (14.4516.18) | 15.68 (14.8216.54) | 16.05 (15.0917.00) | 0.27 |
| Model 2 | 15.25 (14.4016.10) | 15.70 (14.8316.57) | 16.10 (15.1317.08) | 0.20 |
| Model 3 | 15.36 (14.5116.21) | 15.58 (14.7416.43) | 16.10 (15.1317.07) | 0.27 |
| *Current non-smokers (N=711)* | | | | |
| Model 1 | 14.17 (13.3215.02) | 14.47 (13.7315.20) | 14.45 (13.6115.29) | 0.64 |
| Model 2 | 14.10 (13.2614.93) | 14.51 (13.7915.24) | 14.47 (13.6315.31) | 0.54 |
| Model 3 | 14.10 (13.2714.93) | 14.48 (13.7715.20) | 14.50 (13.6715.33) | 0.51 |

Adjusted means for NMD with 95% CIs are given. P_trend_: p for linear trend; NMD, nitrate-mediated dilation. Model 1: adjusted for time between core and NMD examination, age (10-year-categories) and sex; Model 2: Model 1 plus school education (three categories) and smoking status (three categories); Model 3 – fully adjusted model: Model 2 plus diabetes, waist circumference, High-density lipoprotein cholesterol, Low-density lipoprotein cholesterol, and hypertension. None of the associations was significant.
