# Supplementary material for: Periodontitis Is Associated with Endothelial Dysfunction in a General Population: A Cross-Sectional Study
Source: PLoS One. 2013 Dec 26;8(12):e84603. doi: 10.1371/journal.pone.0084603 (PMC3873439; doi:10.1371/journal.pone.0084603)
Supplement: Table S4 — Association between mean clinical attachment loss (tertiles, exposure) and NMD (dependent variable). (DOCX) [file pone.0084603.s004.docx]

Table S4. Association between mean clinical attachment loss (tertiles, exposure) and NMD (dependent variable).

|  | Mean clinical attachment loss | | |  |
| --- | --- | --- | --- | --- |
|  | 0-1.14 mm (ref.) | 1.16-2.58 mm | 2.58-11.86 mm | P_trend_ |
| *All subjects (N=904)* | | | | |
| Model 1 | 14.04 (13.23; 14.86) | 14.82 (14.10; 15.56) | 15.04 (14.33; 15.75) | 0.10 |
| Model 2 | 13.82 (13.01; 14.63) | 14.76 (14.03; 15.49) | 15.28 (14.54; 16.02) * | 0.02 |
| Model 3 | 13.94 (13.13; 14.76) | 14.68 (13.96; 15.39) | 15.26 (14.53; 15.99) * | 0.03 |
| *Subjects without antihypertensive medication (N=578)* | | | | |
| Model 1 | 15.13 (14.20; 16.06) | 15.98 (15.09; 16.88) | 16.23 (15.22; 17.25) | 0.14 |
| Model 2 | 14.96 (14.03; 15.89) | 16.09 (15.19; 17.00) | 16.32 (15.25; 17.38) | 0.08 |
| Model 3 | 15.16 (14.22; 16.09) | 16.00 (15.12; 16.89) | 16.17 (15.12; 17.23) | 0.18 |
| *Current non-smokers (N=670)* | | | | |
| Model 1 | 13.84 (12.87; 14.81) | 14.45 (13.65; 15.26) | 15.28 (14.44; 16.11) * | 0.04 |
| Model 2 | 13.70 (12.74; 14.66) | 14.40 (13.61; 15.20) | 15.43 (14.56; 16.31) * | 0.01 |
| Model 3 | 14.09 (13.48; 14.71) | 14.48 (14.00; 14.95) | 15.12 (14.45; 15.79) * | 0.02 |

Adjusted means for NMD with 95% CIs are given. P_trend_: p for linear trend; NMD, nitrate-mediated dilation. Model 1: adjusted for time between core and NMD examination, age (10-year-categories) and sex; Model 2: Model 1 plus school education (three categories) and smoking status (three categories); Model 3 – fully adjusted model: Model 2 plus diabetes, waist circumference, High-density lipoprotein cholesterol, Low-density lipoprotein cholesterol, and hypertension. * p<0.05 versus reference category (ref.)
