# Supplementary material for: Periodontitis Is Associated with Endothelial Dysfunction in a General Population: A Cross-Sectional Study
Source: PLoS One. 2013 Dec 26;8(12):e84603. doi: 10.1371/journal.pone.0084603 (PMC3873439; doi:10.1371/journal.pone.0084603)
Supplement: Table S5 — Association between hs-CRP (tertiles, exposure) and NMD (dependent variable). (DOCX) [file pone.0084603.s005.docx]

Table S5. Association between hs-CRP (tertiles, exposure) and NMD (dependent variable).

|  | hs-CRP | | |  |
| --- | --- | --- | --- | --- |
|  | 0.17-0.816 mg/l (ref.) | 0.82-2.07 mg/l | 2.09-29.1 mg/l | P_trend_ |
| *All subjects (N=951)* | | | | |
| Model 1 | 15.17 (14.45; 15.90) | 13.86 (13.20; 14.53) ** | 14.49 (13.79; 15.18) | 0.21 |
| Model 2 | 15.18 (14.45; 15.91) | 13.80 (13.15; 14.46) ** | 14.54 (13.85; 15.24) | 0.25 |
| Model 3 | 14.74 (14.00; 15.49) | 13.85 (13.20; 14.51) | 14.90 (14.19; 15.60) | 0.73 |
| *Subjects without antihypertensive medication (N=597)* | | | | |
| Model 1 | 16.21 (15.38; 17.05) | 15.81 (13.94; 15.67) * | 15.93 (14.96; 16.89) | 0.61 |
| Model 2 | 16.28 (15.44; 17.12) | 14.70 (13.84; 15.56) * | 15.97 (15.07; 16.93) | 0.59 |
| Model 3 | 15.89 (15.03; 16.75) | 14.81 (13.95; 15.67) | 16.30 (15.32; 17.28) | 0.58 |
| *Current non-smokers (N=711)* | | | | |
| Model 1 | 14.87 (14.05; 15.70) | 13.81 (13.05; 14.58) | 14.44 (13.63; 15.24) | 0.48 |
| Model 2 | 14.90 (14.08; 15.72) | 13.72 (12.96; 14.48) * | 14.50 (13.69; 15.30) | 0.54 |
| Model 3 | 14.50 (13.65; 15.35) | 13.71 (12.96; 14.47) | 14.86 (14.04; 15.69) | 0.54 |

Adjusted means for NMD with 95% CI are given. P_trend_: p for linear trend; NMD, nitrate-mediated dilation. Model 1: adjusted for time between core and FMD examination, age (10-year-categories), and sex; Model 2: Model 1 plus school education (three categories) and smoking status (three categories); Model 3 – fully adjusted model: Model 2 plus diabetes, waist circumference, High-density lipoprotein cholesterol, Low-density lipoprotein cholesterol, and hypertension. * p<0.05, ** p<0.01 versus reference category (ref.)
